# Effects of Trazodone on Sleep Quality and Cognitive Function in Arteriosclerotic Cerebral Small Vessel Disease Comorbid With Chronic Insomnia

**DOI:** 10.3389/fpsyt.2020.00620

**Published:** 2020-06-30

**Authors:** Jihui Wang, Sanxin Liu, Chongbang Zhao, Hongying Han, Xiaodong Chen, Jiong Tao, Zhengqi Lu

**Affiliations:** ^1^Department of Psychiatry, The Third Affiliated Hospital, Sun Yat-sen University, Guangzhou, China; ^2^Department of Neurology, The Third Affiliated Hospital, Sun Yat-sen University, Guangzhou, China

**Keywords:** trazodone, small vessel disease, sleep, cognitive function, polysomnography

## Abstract

**Background:**

Chronic insomnia is common in patients with arteriosclerotic cerebral small vessel disease (CSVD) and aggravates the cognitive impairment caused by CSVD. Low-dose trazodone is effective in treating insomnia, but it is unclear whether it can also improve cognitive function in CSVD patients. This study was performed to explore the effects of trazodone on sleep quality and cognitive function in CSVD comorbid with chronic insomnia.

**Methods:**

This was a randomized, double-blind, placebo-controlled pilot study. Forty patients suffering from arteriosclerotic CSVD and insomnia were recruited from an outpatient clinic. Participants were randomized individually to receive either trazodone (study group) or a placebo (control group) for 4 weeks. The primary outcome was the cognitive score on the Montreal Cognitive Assessment scale (MoCA). Secondary outcomes included sleep parameters measured with polysomnography (PSG) and the Pittsburgh Sleep Quality Index.

**Results:**

Trazodone caused significantly better improvements in concentration and recall abilities, measured with MoCA, as well as in PSG parameters such as sleep efficiency, N3 sleep ratio, and sleep continuity than the placebo, with no significant differences in the occurrence of side effects. The improvement of sleep quality was correlated with increased concentration and recall abilities.

**Conclusions:**

A low dose of trazodone seems acceptable and effective in reducing insomnia severity and improving concentration and recall abilities in this pilot study. The improvement of cognition could be achieved by alleviation of insomnia severity. Considering the high incidence of insomnia in CSVD patients, the results of this preliminary study support the use of low-dose trazodone to deal with insomnia and cognitive impairment in CSVD.

## Introduction

Cerebral small vessel disease (CSVD) refers to the syndrome of clinical, cognitive, imaging, and pathological manifestations caused by various small vascular diseases (1), among which arteriosclerotic CSVD is the most common type (1). CSVD accounts for 50–70% of vascular cognitive impairment cases and 45% of dementia cases and results in a heavy social burden (2).

Chronic insomnia, the most common sleep disorder in elderly individuals, is often comorbid with physical diseases including cerebral vascular disease (3). An earlier study by our team found that the proportion of individuals with persistent insomnia among CSVD patients (54%) (4) was far higher than that among the general elderly population (12–20%) (5). The sleep patterns of these patients are characterized by increased sleep fragmentation, decreased sleep efficiency, and a reduced slow-wave sleep (SWS) ratio (4). Insomnia is frequently associated with cognitive decline and is thought to be partly responsible for the pathological progression of several neurodegenerative diseases (6). According to the results of our prophase research, the disruption of sleep continuity caused by insomnia can aggravate the impairment of executive function and memory in CSVD patients (4), suggesting that treating insomnia might be a potential target for improving the cognitive function of CSVD patients. Evidence indicates that the effective treatment of insomnia helps to alleviate comorbid disorders (7). Medication and psychotherapy are both effective methods for treating chronic insomnia (8). However, cognitive and behavioral therapy is not always an option due to the lack of well-trained therapists and the long treatment sessions required.

Trazodone, a second-generation triazolopyridine antidepressant, is approved for the treatment of depression. Because of its dose-dependent pharmacologic actions, low dose trazodone (25–150 mg) is effective in blocking histamine 1 (H_1_), 5 hydroxytryptamine 2A (5-HT_2A_), and *α*1-adrenergic receptors and is more often used as a sedative for treating insomnia (9). Several studies have demonstrated that trazodone is helpful for improving nocturnal sleep maintenance without a hangover effect due to the relatively short half-value period (3–6 h) (9). Moreover, trazodone may help improve cognitive function by increasing the concentration of 5-HT in the synaptic space. This theory is based on the finding that cognitive impairment is related to 5-HT deficiency and the normalization of 5-HT function contributes to the improvement of cognitive function (10). In the past, studies on the cognitive effects of trazodone focused on the side effects of the drug and the results were inconsistent. Ip EJ (2013) evaluated the cognitive driving ability in healthy adults after a single dose (100 mg) of trazodone and found that the number of individual impairment clues increased 2 h after drug administration (11). Sasada K (2013) evaluated the effect of mirtazapine and trazodone (25 mg) on cognitive function in healthy men after 9 days of treatment and reported that trazodone did not affect cognitive function (12). Roth AJ (2011) used trazodone (50 mg) to treat primary insomnia, and the results showed that trazodone improved sleep quality but caused small impairments in short-term memory after 1 week of treatment (13). The observation periods for the above studies were generally short, and the participants were not patients with cognitive impairment. To the best of our knowledge, few studies have assessed the effects of trazodone on cognitive impairment in individuals with CSVD comorbid with persistent insomnia. This pilot study was conducted to explore the clinical efficacy and cognitive remediation of trazodone (50 mg dose) treatment for these patients. We hypothesized that trazodone would improve cognitive function in addition to relieving insomnia.

## Methods

### Study Design

This was a double-blinded, placebo-controlled pilot study with a 4-week follow-up period. Participants were randomly assigned to the study group (trazodone) or the control group (placebo). Informed consent was required for all participants. The ethical committee of the Third Affiliated Hospital of Sun Yat-sen University approved the trial (Ethical code: [2019]02-414-01).

### Inclusion Criteria

1. Aged 40 to 70.
2. Presence of traditional vascular risk factors, such as hypertension, diabetes, hypercholesterolemia, or smoking
3. Evidence of the presence of CSVD imaging markers (14): lacunar infarcts, moderate to severe white matter hyperintensities (Fazekas ≥ 2) (15), visible perivascular spaces, and cerebral microbleeds
4. Presence of cognitive impairment [total Montreal Cognitive Assessment (MoCA) score < 23]. In accordance with domestic research in China (16), a MoCA score of 23 was defined as the cutoff value to distinguish cognitive impairment in CSVD patients
5. CSVD and other somatic disorders had been effectively treated
6. Met the clinical diagnostic criteria for persistent insomnia in the Diagnostic and Statistical Manual of Mental Disorders, 5th Edition (DSM-V) (17)
7. Pittsburgh Sleep Quality Index (PSQI) ≥ 6
8. Hamilton Depression Scale (HAMD)-17 score < 17
9. Signed the written informed consent form

### Exclusion Criteria

1. Patients who were intolerant of or allergic to trazodone
2. Patients taking medicine that interferes with sleep or cognition within 2 weeks before enrollment
3. Patients with other types of CSVD apart from arteriolosclerosis
4. Evidence of main artery disease or embolic cerebral infarction
5. Presence of DSM-V sleep disorders other than insomnia, serious mental illness such as psychotic disorders, major depressive disorder, bipolar disorders, dementia, or substance-related and addictive disorders
6. Patients unable to complete the data collection process
7. Night-shift workers

### Recruitment

Patients seeking medical advice because of cognitive impairment, abnormal gait, dizziness, dysphagia, dysuria, or emotional distress were recruited through the outpatient clinic of the Third Affiliated Hospital of Sun Yat-sen University and physician referrals. Potentially eligible participants received clinical consultation, a structured interview, and physical examination by an experienced neurologist, then they were asked to complete brain magnetic resonance (MR) scans, polysomnography (PSG), and questionnaires. The participants who met the inclusion criteria were enrolled.

### Randomization

Participants were randomized with a ratio of 1:1 by drawing cards in a dark box.

### Administration of Study Medication

Trazodone (50 mg, Mei Shi Chemical Pharmaceutical Company, Taiwan) tablets and placebo pills in empty capsules were prepared. We chose a dose of 50 mg because this dose is often used for sedation and hypnosis (18). The patients took the medication orally once daily, half an hour before bedtime. If the patients could not tolerate a dose of 50 mg, they were allowed to decrease the dose to 25 mg/day for 3 days and return to 50 mg/day on the fourth day. Patients who could not tolerate a dose of 25 mg were withdrawn from the trial. The medications that the patients were taking to treat general physical problems were allowed provided the treatment regimen was stable during the 2-week period before enrollment.

### Brain MR Scan

MR scans were performed using a 3.0-T MR scanner (General Electric). The sequences for three-dimensional time of flight MR angiography, MR imaging T1 Fluid Attenuated Inversion Recovery (Flair), T2 Flair, T2-weighted images, and susceptibility weighted images were collected. Images were coded by an experienced radiologist for each CSVD marker. A CSVD burden score ranging from 0 to 4 was calculated based on the presence of each marker (19).

### Outcome Measurement

#### Primary Outcome

##### MoCA-Beijing Version

The MoCA test was used to assess several cognitive domains: visuospatial/executive functions, naming, concentration, language, abstraction, short-term memory recall, and orientation, with an aggregate score ranging from 0 to 30. One point was added if the educational level of the patient was less than 12 years (20).

#### Secondary Outcomes

##### PSG

PSG was recorded three times (twice at baseline and once at posttreatment) at a sleep center (PSG manufacturer: Cadwell Laboratories. Inc, product model: Easy III). The first night’s PSG was performed for patients to become accustomed to the sleep center and only the data from the latter two monitoring sessions were included in the analysis. Patients were required to arrive at the sleep center at 9:00 p.m. Monitoring started at 10:00 p.m. and lasted for at least 8 h. PSG recordings included electroencephalogram, chin movements, leg movements, eye movements, electrocardiogram, oxygen saturation, and chest and abdominal wall movement. PSG data were analyzed according to the standard criteria (21). The following PSG parameters were collected as we reported in a previous study (4): sleep onset latency (SOL), total sleep time (TST), sleep efficiency (SE), wake after sleep onset (WASO), proportion of sleep stages (N1, N2, N3, and rapid eye movement [REM]), apnea-hypopnea index (AHI), and arousal index (ArI).

##### PSQI–Chinese Version

Sleep quality was measured using PSQI, which consists of 17 items with seven components. Each component is scored from 0 to 3 and the global score ranges from 0 to 21 (22).

##### Epworth Sleepiness Scale (ESS)

Daytime sleepiness was measured with ESS, which consists of eight items. Each item is scored from 0 to 3 and the global score ranges from 0 to 24 (23).

##### Fatigue Scale (FS-14)

Daytime fatigue was measured with FS-14, which consists of 14 items. Each item is scored from 0 to 1 and the global score ranges from 0 to 14 (24).

##### The Hamilton Depression Scale (HAMD)-17

Depressive symptoms were measured with HAMD-17, which consists of 17 items. The global score ranges from 0 to 54 and the cutoff point to identify moderate depression is 17 points (25).

##### The Hamilton Anxiety Scale (HAMA)

Anxiety symptoms were measured with HAMA, which consists of 14 items with a global score ranging from 0 to 56. Scores over 14 indicate moderate to severe anxiety (26).

##### Safety and Tolerability

The patients were encouraged to report any adverse events during the study. The treatment emergent symptom scale (TESS) was used to assess the adverse events related to several organ systems and abnormal results in the laboratory examinations. TESS consists of 34 items with the score for each item ranging from 0 to 4 (27).

### Statistical Analysis

SPSS (version 20.0) was used for statistical analysis. The demographic data and clinical characteristics were described as the mean ± standard deviations or proportions as needed. The PSG-measured parameters, sleep-related questionnaires, and cognitive scores were analyzed using two-way ANOVA for repeated measures with the main effects as the group (trazodone *vs* placebo) and time (pre or posttreatment) and the interaction effect as group × time. The relationship between sleep improvement and cognitive improvement was analyzed using Pearson correlation analyses. Classification data was analyzed with χ2 tests or Fisher Exact statistics where appropriate. All tests were two-tailed, and all analyses were defined as significant when *P* < 0.05.

## Results

### Demographic and Clinical Characteristics of the Participants

**Figure 1:**
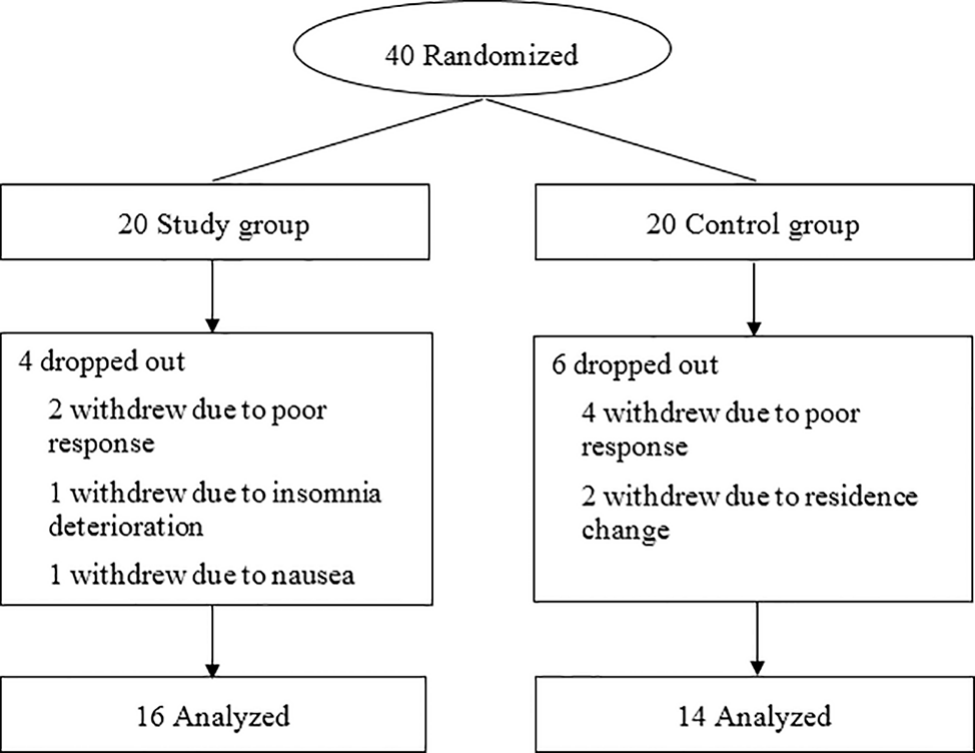
Patient flow chart.

The recruitment began on February 1, 2019 and ended on December 31, 2019. Forty patients were randomly assigned to the trazodone and placebo groups, among which 30 patients completed all follow-ups and evaluations (16 in the study group and 14 in the control group). There was no significant difference between the patients who dropped out (n  =  10) and those who completed the follow-ups (n  =  30) in age (62.74 *vs* 62.89 years), proportion of male patients (60 *vs* 50%), baseline PSQI scores (10.52 *vs* 10.87), and MoCA scores (17.85 *vs* 17.36) (*P* > 0.05). The patient flow chart is shown in **Figure 1**, while the demographic and clinical data are shown in **Table 1**.

**Table 1:** Demographic and clinical data of the participants.

Variables	Study group (n = 16)	Control group (n = 14)	t/χ^2^	P
Age, year	62.65 ± 12.57	63.14 ± 11.65	0.388	0.701
Body mass index	21.59 ± 4.85	22.24 ± 2.24	0.569	0.574
Male, n (%)	7 (43.75%)	8 (57.14%)	0.536	0.464
Single, n (%)	2 (12.50%)	3 (21.43%)	–	0.642^a^
Education ≤ 12 years, n (%)	11 (68.75%)	10 (71.43%)	0.026	0.873
Hypertension, n (%)	7 (43.75%)	6 (42.86%)	0.002	0.961
Diabetes, n (%)	5 (31.25%)	6 (42.86%)	0.433	0.510
Hypercholesterolemia, n (%)	10 (62.50%)	8 (57.14%)	0.089	0.765
Ischemic heart disease, n (%)	4 (25.00%)	4 (28.57%)	–	0.574^a^
Smokers, n (%)	3 (18.75%)	4 (28.57%)	–	0.675^a^
CSVD MR burden	1.82 ± 1.04	1.75 ± 1.24	0.764	0.452
Insomnia duration, month	18.24 ± 14.54	20.54 ± 12.35	1.119	0.272
Insomnia severity index	17.3 ± 2.43	17.5 ± 2.24	0.323	0.748

a Fisher exact test.

### Comparisons of Cognitive Scores Between the Two Groups

At baseline, the differences between the groups with regard to the total MoCA scores and factor scores were not statistically significant (*P* > 0.05). After treatment, the factor scores for the concentrations and recall in the trazodone group increased compared to those at baseline (*P* < 0.05). Repeated measures ANOVA showed that trazodone treatment resulted in greater improvement in the concentration and recall abilities than the placebo (*P* < 0.05), as shown in **Table 2**. The group × time interaction and the group and time main effects were as follows:

Concentration (F_group__×__time_ = 2.385, *P* = 0.034; F_group_ = 0.757, *P* = 0.585; F_time_ = 2.684, *P* = 0.027)

Recall (F_group__×__time_ = 5.854, *P* < 0.001; F_group_ = 3.595, *P* = 0.010; F_time_ = 4.521, *P* < 0.001)

**Table 2:** Comparison of cognitive scores between the two groups.

Measures	Study group (SG, n = 16)		Control group (CG, n = 14)		SG-CG (SE)^(2)^ [95% CI]
	Pre/post Mean (SD)	Difference (SE)^(1)^ 95% CI	Pre/post Mean (SD)	Difference (SE)^(1)^ [95% CI]	
Total score	17.03 (4.53)/ 19.30 (4.62)	−2.07 (0.42) −4.79 to 0.65	17.70 (4.29)/ 18.01 (4.73)	−0.31 (0.08) −0.65 to 0.04	−1.76 (0.44) -3.77 to 0.25
Visuospatial/executive	2.14 (1.22)/2.32 (1.17)	−0.18 (0.11) −0.38 to 0.02	2.22 (1.25)/ 2.28 (1.27)	−0.06 (0.03) −0.18 to 0.06	−0.12 (0.02) −0.28 to 0.04
Naming	2.14 (0.84)/2.17 (0.79)	−0.03 (0.01) −0.07 to 0.01	2.11 (0.81)/ 2.15 (0.75)	−0.04 (0.01) −0.11 to 0.03	0.01 (0.01) −0.04 to 0.06
Concentration	4.41 (1.57)/5.33 (1.45)	−0.92 (0.12) −1.41 to −0.43	4.86 (1.46)/ 4.91 (1.48)	−0.05 (0.02) −0.08 to −0.02	−0.87 (0.07) −1.38 to −0.36
Language	1.72 (1.14)/1.78 (1.08)	−0.06 (0.02) −0.11 to −0.01	1.74 (1.10)/ 1.76 (1.05)	−0.02 (0.01) −0.05 to 0.01	−0.04 (0.01) −0.10 to 0.02
Abstraction	0.68 (0.48)/0.72 (0.46)	−0.04 (0.01) [−0.09 to 0.01]	0.67 (0.51)/ 0.66 (0.52)	0.01 (0.02) [−0.04 to 0.06]	−0.05 (0.01) −0.13 to 0.03
Recall	1.52 (1.03)/2.24 (1.12)	−0.72 (0.21) [−1.15 to −0.29]	1.54 (0.95)/ 1.63 (1.01)	−0.09 (0.02) [−0.21 to 0.03]	−0.63 (0.07) −0.84 to −0.42
Orientation	4.52 (0.84)/ 4.71 (0.75)	−0.19 (0.22) [−0.39 to 0.01]	4.55 (0.95)/ 4.62 (0.74)	−0.07 (0.02) [−0.17 to 0.03]	−0.12 (0.03) −0.41 to 0.17

^(1)^Indicates mean change between baseline and posttreatment evaluation.

^(2)^Indicates differences in mean change between the study group and the control group.

### Comparison of PSG Parameters Between the Two Groups

At baseline, there was no significant difference in the PSG parameters between the groups (*P* > 0.05). After treatment, the study group exhibited an increase in SE and N3 sleep ratio as well as a decrease in WASO, N1 sleep ratio, and ArI compared with the values at baseline (*P* < 0.05). Repeated measures ANOVA showed that trazodone induced greater improvements than placebo in PSG-measured SE, WASO, N1, N3, and ArI, *P* < 0.05, as shown in **Table 3**. The group × time interaction and the group and time main effects were as follows:

SE (F_group__×__time_ = 2.426, *P* = 0.031; F_group_ = 2.328, *P* = 0.057; F_time_ = 4.632, *P* < 0.001)

WASO (F_group__×__time_ = 6.452, *P* < 0.001; F_group_ = 3.983, *P* = 0.008; F_time_ = 5.673, *P* < 0.001)

N1% (F_group__×__time_ = 4.953, *P* < 0.001; F_group_ = 5.248, *P* < 0.001; F_time_ = 7.358, *P* < 0.001)

N3% (F_group__×__time_ = 3.954, *P* = 0.009; F_group_ = 4.672, *P* < 0.001; F_time_ = 3.428, *P* = 0.016)

ArI (F_group__×__time_ = 2.984, *P* = 0.022; F_group_ = 3.014, *P* = 0.021; F_time_ = 3.584, *P* = 0.011)

**Table 3:** Comparisons of sleep quality between the two groups.

PSG	Study group (SG, n = 16)		Control group (CG, n = 14)		SG-CG (SE)^(2)^ 95% CI
	Pre/post Mean (SD)	Difference (SE)^(1)^ 95% CI	Pre/post Mean (SD)	Difference (SE)^(1)^ 95% CI	
SOL, min	35.76(16.82)/ 29.41(18.56)	6.25(5.25) 3.55 to 8.95	34.14(14.64)/32.82(16.43)	1.32(1.14) −0.51 to 3.15	4.92(1.87) −1.22 to 11.06
TST, min	375.56(58.46)/ 392.48(64.55)	−16.92(6.33) −33.95 to 0.11	354.57(64.85)/361.20(60.40)	−6.63(2.6) −14.55 to 1.29	−10.29 (3.36) −25.53 to 4.95
SE, %	74.55(14.54)/ 81.67(11.87)	−7.12(1.53) −11.45 to −2.79	75.52(17.5)/75.88(22.4)	−0.36(0.13) −0.76 to 0.04	−6.76(2.1) −11.46 to −2.06
WASO, min	61.56(37.56)/ 42.42 (20.88)	19.14(8.53) 14.58 to 23.70	58.63(34.40)/52.46(25.87)	6.17(2.53) 3.52 to 8.82	12.97(4.53) 8.44 to 17.51
N1, %	13.91 (5.66)/ 9.44(4.96)	4.47(2.13) 2.90 to 6.04	13.65 (4.73)/13.12 (5.24)	0.53(0.22) −0.35 to 1.41	3.94(0.7) 2.11 to 5.77
N2, %	61.10 (11.38)/ 59.72(13.59)	1.38(1.12) −0.37 to 3.03	60.52 (12.65)/ 61.31 (11.56)	−0.79(0.12) −1.73 to 0.15	2.17 (0.22) −0.32 to 4.66
N3, %	10.63 (5.35)/15.37(4.93)	−4.74(3.22) −5.94 to −3.54	11.32 (6.32)/ 10.64 (6.87)	0.68(0.25) −0.24 to 1.50	−5.42(1.12) −7.22 to −1.62
REM, %	14.36 (6.43)/15.(7.22)	−1.11(0.32) −2.77 to 0.55	14.51 (7.25)/ 14.93 (7.15)	−0.42(0.24) −0.91 to 0.06	−0.69(0.12) −1.77 to 0.39
ArI	26.92 (9.45)/ 22.51(11.57)	4.41(1.12) 3.10 to 5.72	26.53(10.57)/26.37(11.1)	0.16(0.03) −0.14 to 0.46	4.25(0.82) 3.14 to 5.36
AHI	4.12(1.51)/ 3.92(1.72)	0.20(0.03) −0.06 to 0.46	4.24(1.41)/4.12(1.35)	0.12(0.02) −0.03 to 0.27	0.08(0.01) −0.04 to 0.20
AI	1.74(0.74)/ 1.66(0.87)	0.08(0.03) −0.11 to 0.27	1.76(0.71)/1.62(0.75)	0.14(0.06) −0.10 to 0.34	−0.06(0.01) −0.17 to 0.05

SOL, sleep onset latency; TST, total sleep time; SE, sleep efficiency; WASO, wake after sleep onset; REM, rapid eye movement sleep; N, non-REM; ArI, arousal index; AHI, apnea and hypopnea index; AI, apnea index.

^(1)^Indicates mean change between baseline and post-treatment evaluation.

^(2)^Indicates differences in mean change between the study group and the control group.

### Comparison of Scale Measures Between the Two Groups

At baseline, there was no significant difference in the scale scores between the groups (*P* > 0.05). After the treatment, the study group had lower scores in PSQI, ESS, and HAMA compared with those at baseline (*P* < 0.05). Repeated measures ANOVA showed that trazodone caused greater improvements than the placebo in PSQI, ESS, and HAMA (*P* < 0.05), as shown in **Table 4**. The group × time interaction and the group and time main effects were as follows:

PSQI (F_group__×__time_ = 4.843, *P* < 0.001; F_group_ = 1.018, *P* = 0.295; F_time_ = 5.453, *P* < 0.001)

ESS (F_group__×__time_ = 7.035, *P* < 0.001; F_group_ = 3.352, *P* = 0.018; F_time_ = 4.732, *P* < 0.001)

HAMA (F_group__×__time_ = 5.268, *P* < 0.001; F_group_ = 2.367, *P* = 0.046; F_time_ = 2.374, *P* = 0.041)

**Table 4:** Comparison of scale measures between the two groups.

Scales	Study group (SG, n = 16)		Control group (CG, n = 14)		SG-CG (SE)^(2)^ 95% CI
	Pre/post Mean (SD)	Difference (SE) ^(1)^ 95% CI	Pre/post Mean (SD)	Difference (SE)^(1)^ 95% CI	
PSQI	11.57 (2.13)/ 7.33 (1.66)	4.24 (1.14) 3.21 to 5.27	10.17 (1.93)/ 9.32 (1.42)	0.85 (0.25) −0.22 to 1.92	3.39 (1.42) 1.83 to 5.95
ESS	6.87 (3.82)/ 4.12 (2.74)	2.75 (0.44) 0.95 to 4.55	6.83 (3.12)/ 6.46 (2.25)	0.37 (0.16) −0.14 to 0.88	2.38 (0.12) 1.01 to 3.75
FS-14	6.95 (2.72)/ 6.38 (2.57)	0.57 (0.24) −0.61 to 1.75	6.32 (2.70)/ 6.46 (1.95)	−0.14 (0.04) −0.32 to 0.04	0.71 (0.15) −0.14 to 1.56
HAMD	8.13 (5.15)/ 6.65 (2.32)	1.48 (0.12) 0.34 to 2.62	7.94 (4.12)/ 7.36 (4.42)	0.28 (0.09) −0.22 to 0.78	1.70 (0.11) −0.22 to 3.62
HAMA	11.92 (4.91)/ 8.04 (3.50)	3.88 (1.15) 1.24 to 6.52	12.13 (4.68)/ 11.72 (4.84)	0.41 (0.12) −0.09 to 0.91	3.47 (0.88) 1.26 to 5.68

PSQI, Pittsburgh Sleep Quality Index; ESS, Epworth Sleepiness Scale; FS-14, Fatigue Scale; HAMD, Hamilton Depression Scale; HAMA, Hamilton Anxiety Scale.

^(1)^Indicates mean change between baseline and post-treatment evaluation.

^(2)^Indicates differences in mean change between the study group and the control group.

### Correlation Analysis of Sleep Improvement and Cognitive Improvement

The improvement of concentration and recall was positively related to the improvement of PSQI, ESS, HAMA, N3 ratio, WASO, and SE as shown in **Table 5**.

**Table 5:** Correlation analysis of sleep improvement and cognitive improvement.

Variables	PSQI		ESS		HAMA		ArI		N3, %		WASO		SE	
	*r*	*p*	*R*	*p*	*r*	*p*	*r*	*p*	*r*	*p*	*r*	*p*	*r*	*p*
Concentration	0.291	0.033	0.466	<0.001	0.375	0.005	0.175	0.206	0.318	0.019	0.423	0.001	0.282	0.03
Recall	0.400	0.003	0.566	<0.001	0.332	0.014	0.298	0.029	0.339	0.012	0.269	0.049	0.545	<0.001

PSQI, Pittsburgh Sleep Quality Index; ESS, Epworth Sleepiness Scale; HAMA, Hamilton Anxiety Scale. ArI, arousal index; N, non-REM; WASO, wake after sleep onset; SE, sleep efficiency.

### Comparison of Adverse Events Between the Two Groups

The participants in this study tolerated the treatment well. All the adverse events reported by the participants were mild. We did not calculate the mean score for each item in TESS because most of them were reported as zero. The most frequently reported adverse reactions were insomnia deterioration, akathisia, nausea, loss of appetite, dizziness, and headache. During the whole trial, no serious abnormal laboratory results related to the trial were reported. As shown in **Table 6**, we did not observe significant differences in the occurrence of side effects between the two groups.

**Table 6:** Comparison of side effects between the two groups.

Groups	Insomnia deterioration	Akathisia	Nausea	Loss of appetite	Dizziness	Headache
Study group	2 (12.5%)	2 (12.5%)	2 (12.5%)	4 (25.0%)	3 (18.8%)	1 (6.2%)
Control group	4 (28.6%)	3 (21.4%)	1 (7.1%)	2 (14.3%)	4 (28.6%)	2 (14.3%)
Fisher Exact, *P*	0.378	0.642	1.000	.657	0.675	0.586

## Discussion

This was a pilot study evaluating the effects of trazodone on insomnia and cognitive impairment in patients with CSVD comorbid with persistent insomnia. Potential confounders were controlled because patients with dementia, major depression, and breathing-related sleep disorders were excluded, which made the results of this study more convincing.

The safety and tolerability of medication were the primary consideration in the study since most patients suffering from CSVD are middle-aged and elderly. The patients in the present study tolerated trazodone well and did not report any serious adverse events. The discontinuation rates were almost the same in the two groups. All adverse events reported were mild, such as insomnia deterioration, akathisia, nausea, loss of appetite, dizziness, and headache, which were roughly similar to the results of previous studies (28, 29). As shown in the results, three CSVD patients treated with trazodone reported dizziness, which should receive more attention because trazodone may increase the risk of postural hypotension due to its antagonistic effect on the *α*1-adrenergic receptor. No patients showed any signs of hypotension based on the blood pressure monitoring, excluding the possibility of dizziness caused by postural hypotension.

The efficacy of trazodone has been previously demonstrated in several studies for different kinds of sleep disorders, such as primary insomnia (13, 30), insomnia associated with dementia (31), and obstructive sleep apnea syndrome (32). However, this is the first study to evaluate the efficiency of trazodone in CSVD comorbid with persistent insomnia. The results of the PSG test showed that trazodone decreased WASO and ArI while increasing sleep efficiency and N3 sleep ratio. This indicated that trazodone improved sleep continuity and the ratio of SWS, both of which are considered crucial for memory consolidation (33). In the evaluation of insomnia, subject scale assessment is equally important because the diagnosis of insomnia mainly depends on the subjective feelings of the patient (34). The results of the assessment scales used in the study showed that trazodone improved the sleep quality of patients, as measured with PSQI, which confirmed the efficacy of the drug. Daytime dysfunction, such as fatigue, drowsiness, and mood distress, is also a common symptom of chronic insomnia (34) and CSVD (35), and daytime dysfunction itself is one of the diagnostic criteria for insomnia. In the present study, daytime drowsiness and anxiety decreased following trazodone treatment, which was attributed to the improvement of sleep quality caused by trazodone because both drowsiness (36) and anxiety (37) are closely related to poor nocturnal sleep. With regard to depression, the results showed that the HAMD score significantly decreased in the study group after trazodone treatment, but the difference was not statistically significant compared to the score for the placebo group. The reasons may include: 1. Depression in the patients recruited in the study was not serious based on the requirements of the inclusion criteria; and 2. The low dose of trazodone (50 mg) was not enough to trigger the blockade of the serotonin transporter for the antidepressant effect (18).

CSVD is known to reduce the level of blood flow in the brain, especially in thinner arteries supplying the hippocampal and prefrontal areas, and the resulting cognitive impairment is apparent in patients. In this study, we specifically tested the effects of trazodone on short-term memory and executive tests because of the known involvement of hippocampal and prefrontal areas in sleep-mediated effects *via* trazodone-induced improvements in these cognitive domains. The differences in the research objects as well as the evaluation methods should be taken into consideration when comparing the effects of trazodone on cognitive function reported in different studies. In this study, the mean total score of MOCA at baseline was 17.3 points, which was lower than that reported in previous studies (24–27 points) (38, 39). The reasons for this may include: 1. participation was restricted to CSVD patients with cognitive impairment, confirmed with MoCA (total MoCA score < 23); 2. the education level of the patients was relatively low, with 63% below high school; and 3. the comorbidity with insomnia may also contribute to the aggravation of cognitive impairment. According to the results, the MoCA scores in the study group were not significantly different from those in the control group at baseline. After the treatment, trazodone caused significantly greater improvement in the concentration and recall abilities of the patients compared to the placebo.

Several studies evaluating the effect of trazodone on cognitive function showed mixed results. It was reported that the cognitive reaction speed of healthy people was impaired 2 h after a single trazodone administration (11), while repeated administrations for 9 days had no effect on cognition (12). Another study found that trazodone treatment for 1 week improved sleep quality but slightly damaged short-term memory in patients with primary insomnia. However, the lack of a control group attenuated the strength of this study (13). There have been other studies which reported no adverse effects from trazodone on cognitive function with longer observation periods, such as 2 weeks (29) and more than 1 year (40). Based on the above studies, it is reasonable to conclude that cognitive function may be somewhat damaged in the initial stage due to the sedative effect of trazodone (11), but this dysfunction diminishes gradually with repeated treatment and an increase in the patient’s tolerance to the medication. The results of the present study differed from those of the above studies. In this study, 4-week trazodone treatment improved the cognitive function of CSVD patients instead of impairing it. SWS and sleep continuity are both critical to cognitive function, such as memory consolidation, concentration, alertness, and working memory (41, 42). Since trazodone increased the sleep continuity and the ratio of SWS in the present study, it is reasonable to speculate that the cognitive impairment in CSVD patients could be attenuated along with the relief of insomnia. In this study, we further found that the improvement of concentration and recall abilities was correlated with increased sleep quality with the correlation analysis. The phenomenon of effective insomnia treatment inducing improvements in cognitive function was also observed in previous studies (43, 44). In addition, previous studies showed that daytime sleepiness was associated with cognitive impairment in older people, (45) such as in executive control, information processing speed (46), and delayed recall (47), whereas anxiety was related to cognitive flexibility (48), working memory, and attention ability (49). In the present study, trazodone treatment decreased daytime sleepiness and anxiety in CSVD patients, which could also explain, at least in part, the improvement of cognitive function.

In conclusion, this preliminary study showed that low-dose trazodone increased sleep continuity and SWS and improved concentration and recall ability in individuals with CSVD comorbid with insomnia. Considering the high incidence rate of insomnia in CSVD patients, the results of this study support the off-label use of low-dose trazodone for CSVD patients and provide preliminary evidence for the identification of new therapeutic targets for CSVD. Moreover, side effects from trazodone in elderly individuals are relatively rare according to our observations. We can conclude that a potential beneficial effect in the specific cognitive domains affected by trazodone may be applicable to both cognition-impaired older adults and patients with CVSD.

The limitations of this study include: 1. the study was conducted in a single center in China, limiting the generalizability of the findings to other populations; 2. the sample size of 40 subjects was relatively small for further classification of insomnia; 3. assessment was only conducted at baseline and posttreatment and the dynamic relationship between cognitive function and sleep was not explored; and 4. the follow-up period was relatively short; hence, long-term observation to confirm the persistent effect of trazodone is warranted.

## Data Availability Statement

The datasets generated for this study are available on request to the corresponding authors.

## Ethics Statement

The studies involving human participants were reviewed and approved by the ethics committee of the Third Affiliated Hospital of Sun Yat-sen University. The patients/participants provided their written informed consent to participate in this study.

## Author Contributions

JW carried out the study design and the writing of the thesis. SL, CZ, and HH contributed to the data collection. XC was helpful for conducting analyses. JT and ZL contributed to the study design and supervised the whole experiment. All authors contributed to the article and approved the submitted version.

## Conflict of Interest

The authors declare that the research was conducted in the absence of any commercial or financial relationships that could be construed as a potential conflict of interest.

## Abbreviations

PSQI, Pittsburgh Sleep Quality Index; HAMA, Hamilton Anxiety Scale; HAMD, Hamilton Depression Scale; ESS, Epworth Sleepiness Scale; FS-14, Fatigue Scale; SOL, Sleep Onset Latency; TST: Total Sleep Time; SE, Sleep Efficiency; WASO, Wake After Sleep Onset; NREM, Nonrapid Eye Movement; ArI, Arousal Index.
